# A Success Story: Togo Is Moving toward Becoming the First Sub-Saharan African Nation to Eliminate Lymphatic Filariasis through Mass Drug Administration and Countrywide Morbidity Alleviation

**DOI:** 10.1371/journal.pntd.0002080

**Published:** 2013-04-11

**Authors:** Yao K. Sodahlon, Ameyo Monique Dorkenoo, Kodjo Morgah, Komlan Nabiliou, Kossivi Agbo, Rebecca Miller, Michel Datagni, Anders Seim, Els Mathieu

**Affiliations:** 1 Mectizan Donation Program, Decatur, Georgia, United States of America; 2 Ministère de la Sante, Lomé, Togo; 3 Faculté Mixte de Médecine et de Pharmacie, Université de Lomé, Lomé, Togo; 4 Department of Global Health, Rollins School of Public Health, Emory University, Atlanta, Georgia, United States of America; 5 HDI (Health & Development International), Fjellstrand, Norway; 6 Division of Parasitic Diseases and Malaria, Center for Global Health, Centers for Disease Control and Prevention, Atlanta, Georgia, United States of America; Centers for Disease Control and Prevention, United States of America

## Introduction

Lymphatic filariasis (LF) is a debilitating vector-borne disease predominantly caused by the helminths *Wuchereria bancrofti* and *Brugia malayi* [1], [2]. Endemic in 72 countries, LF is responsible for 5.9 million DALYs lost and is implicated as the second leading cause of disability worldwide by the World Health Organization (WHO) [3]–[5]. Although 70% of those infected do not exhibit symptoms, almost all persons infected have subclinical damage to the lymphatic vessels [6], [7]. An estimated 40 million people are symptomatic with the predominant morbidities associated with LF: lymphedema and/or hydrocele [8].

In recognition of the worldwide burden of LF, in 1997, the World Health Assembly passed the resolution WHA 50.29 calling for collaborative efforts by member states to eliminate the disease as a public health problem [9]. In 2000, the Global Programme to Eliminate Lymphatic Filariasis (GPELF) was formed in response to the WHA resolution and aimed to eliminate the disease by 2020. The program adopted a two-pronged strategy: (1) to interrupt transmission of the causal parasite and (2) to alleviate morbidities associated with the disease [10]. The two pillars of the GPELF's strategy form the basic framework for any successful LF program.

Togo is one of the 34 African countries endemic for lymphatic filariasis and is surrounded by the endemic countries of Benin, Ghana, and Burkina Faso [11]. The National Program to Eliminate Lymphatic Filariasis (NPELF) was founded in 2000 and is one of the few LF programs that address the dual goals of the global elimination program on a national scale. Togo is the first sub-Saharan country to achieve probable interruption of transmission and to move to the post-MDA surveillance phase as defined by the WHO [12]. Here we describe the elements that proved successful in the national strategy to address LF in Togo.

## Assessing the Burden

### Infection

**Figure 1:**
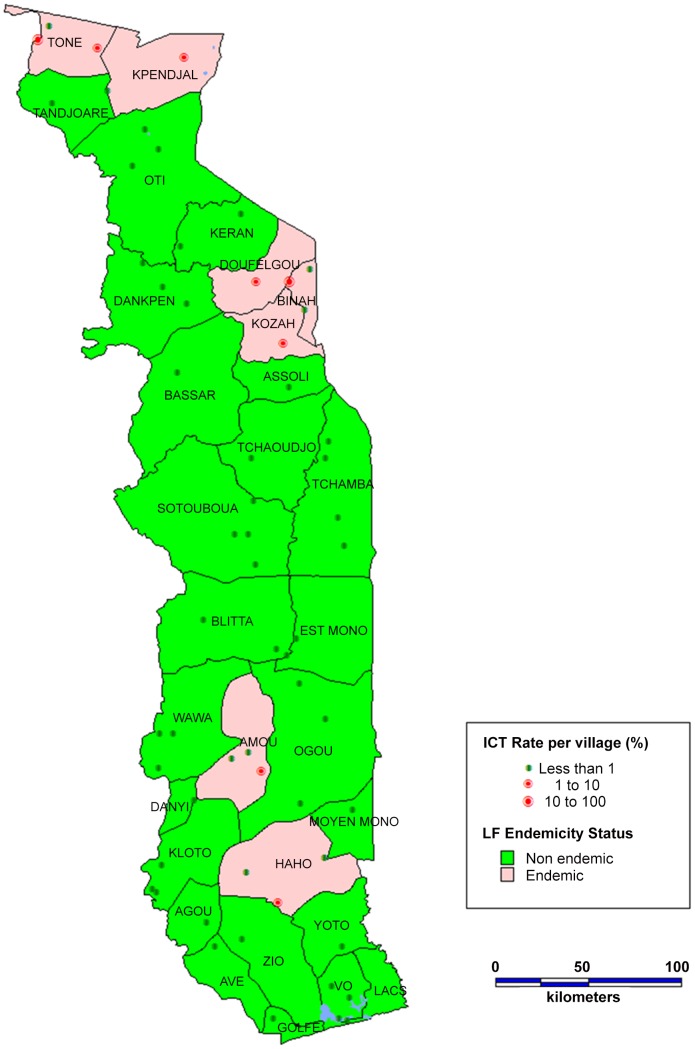
Districts endemic for lymphatic filariasis during the initial mapping.

The Togolese Ministry of Health (MoH) used the Rapid Assessment of Geographical Distribution of Filariasis (RAGFIL) methodology, developed by the Special Programme for Research and Training in Tropical Diseases (TDR), to assess countrywide infection [11], [13]. National mapping was performed in two stages [11]. Thirty-seven villages representing 17 of Togo's 35 health districts (with 5 in Lomé, the capital city) participated in the initial mapping in 1998. In 2000, an additional 24 villages were selected using the WHO's Health Mapper software version 3.0 to ensure that the whole country was covered [11]. The distance between two mapped villages in the final map was never more than 50 km; each selected village represents the state of the transmission in a radius of 25 km. The tests for both stages entailed collection of 100 µl of capillary blood from a person's fingertip and subsequent testing using rapid immunochromatographic tests (ICT) (AMRAD, Townsville, Queensland, Australia). The ICT used is specific for *W. bancrofti* and detects the presence of circulating filarial antigen (CFA) [14]. From the 2009 samples collected in 61 villages, 89 persons (1.8%) were ICT positive. Seven districts were classified as endemic because the prevalence of ICT positivity exceeded the 1% threshold [15], [16] (Figure 1). Several districts along the Togo–Benin border in the south were unexpectedly classified as nonendemic although they were endemic in the past and currently have a high prevalence of LF morbidity [17]. Possibly, LF transmission was interrupted by intensive vector-control methods implemented in the area by the malaria program in the 1970s.

### Morbidity

As accurate estimates of the national burden of LF morbidity are difficult to obtain, Mathieu and others described different methods to obtain LF morbidity prevalence in Togo [18]. Prevalence estimates for lymphedema and hydrocele were calculated based on information collected during LF mapping and from sentinel sites. Morbidity questions were also added onto three 30-cluster drug coverage surveys that were conducted in six of Togo's endemic districts and onto a nationwide malaria bed net coverage survey.

Sentinel site data, cluster survey data, and the malaria bed net coverage survey data detected a hydrocele prevalence of 0.61% (95% CI 0.00–1.41), 0.63% (95% CI 0.20–1.06), and 2.6% (95% CI 1.8–3.4), respectively [19]. These same sources revealed a lymphedema prevalence of 0.80% (95% CI 0.00–1.98), 0.17% (95% CI 0.00–0.34), and 0.6% (95% CI 0.3–0.9).

## Program Implementation

### Mass Drug Administration

Shortly after the mapping, mass drug administration (MDA) campaigns were launched. All of Togo's LF districts are coendemic for onchocerciasis, and the communities with less than 2,000 inhabitants have been undergoing an annual or biannual MDA with ivermectin since the late 1980s [20], [21]. The NPELF modified the distribution system established by the onchocerciasis program through the coadministration of albendazole with ivermectin. The timings of the campaigns were synchronized since LF elimination distributions are required to be organized at the same time in all endemic areas. The geographic coverage in the districts was also expanded to treat villages that were not endemic for onchocerciasis or that had a population above 2,000 inhabitants.

The processes of drug distribution and reporting were facilitated by utilizing Togo's decentralized pyramidal health structure. A single community health worker (CHW) was selected to represent every 300 people in the endemic districts. As the first step toward calculating drug needs, the CHWs visited their respective households prior to each MDA in order to count all inhabitants. The results of the CHWs' annual census were used by the national coordinator to calculate drug needs. The NPELF requested ivermectin (Merck & Co., Inc., White House Station, New Jersey, United States) and albendazole (GlaxoSmithKline, Brentfort, United Kingdom) from the Mectizan Donation Program (MDP). Drug distribution followed the reverse chain of reporting and ended with house-to-house distribution by the CHWs. As children under the age of five are ineligible to receive the treatment, the CHWs used a measuring stick to aid in age estimation and excluded all children with heights below 90 cm [22]. Seriously ill individuals and pregnant women were also excluded from the treatment. The number of people who ingested the drugs was recorded by the CHWs. This data was sent through the appropriate channels back to the NPELF and reported coverage rates calculated.

The first LF MDA was held in the district of Binah in 2000, and within three years, distributions scaled up to all seven endemic districts. Since 2004, reported drug coverage in each district has exceeded 80% of the total population (Table 1). Coverage levels submitted by the CHWs were verified by a coverage survey performed in 2004 (described below). Scaling down started in 2008 and in 2009 when the final MDAs were organized in the districts of Tone and Doufelgou.

**Table 1:** Reported coverage for MDA in endemic districts of Togo (denominator = total population).

	Therapeutic Coverage (%)								
District	MDA1	MDA2	MDA3	MDA4	MDA5	MDA6	MDA7	MDA8	MDA9
**Amou**	74	76	81	86	85	86	85	–	–
**Binah**	81	78	88	86	87	87	84	86	–
**Doufelgou**	80	88	85	85	86	87	87	–	87
**Haho**	79	74	83	82	85	86	85	–	–
**Kozah**	84	87	87	87	87	87	–	–	–
**Kpendjal**	65	89	81	81	83	85	85	85	–
**Tone**	77	75	81	82	84	84	85	84	84

Coverage is calculated out of total population in the area of distribution.

### Lymphedema Management

In collaboration with the CDC and funded by IMA World Health-USAID, the Togo MoH engaged in an innovative approach to implement WHO-recommended lymphedema management techniques on a national scale (including the nonendemic districts). The lymphedema management program began in 2007 by training at least one staff member in each of the 570 health facilities in lymphedema education and care. This was followed by an awareness campaign to inform the population that care for “swollen legs" was available. Patients were trained in self-care by using WHO education materials that were locally adapted, and support structures for patients were created. The program sought to ensure sustainability beyond external funding by being low in cost and by including lymphedema treatment in standard medical training programs.

Over the course of one year, the lymphedema management program scaled up to achieve national coverage. A total of 1,083 patients were enrolled into the program; approximately 30% of whom lived in nonendemic districts. An evaluation of the program, which was conducted three years after the launch, showed that 62% of the persons with a swollen leg, regardless of the cause or severity (reversible swelling was included), were still enrolled in the program.

### Hydrocele Surgeries

Another key element to success was participation in the multicountry West African LF Morbidity Project. Multiple workshops on the current WHO-recommended surgical techniques for hydrocele were held for district-level surgeons on an annual basis from 2005 to 2007 and then again in 2009. Following each workshop, surgical campaigns were conducted [23], along with a series of national campaigns to increase the availability of hydrocelectomies to the affected population.

Since the program's inception, 24 district surgeons have participated in the surgical trainings. During the three campaigns that ran from 2005 to 2006, 257 hydrocelectomies were performed. In 2007 and 2008, 215 hydrocelectomies were performed with additional financial assistance from Health & Development International (HDI).

## Monitoring and Evaluation

The success of the NPELF was partially due to the rigorous monitoring and evaluation system.

### MDA Coverage

Following each annual drug distribution, reported drug coverage was collected using the participation tally submitted by the CHWs. The denominator used was the total population (annual census), which was calculated by the CHWs prior to each MDA. In each, the WHO's goal of 70% drug coverage was achieved, and since 2004, all districts' reported drug coverage has exceeded 80% (see Table 1). In 2004, coverage surveys were conducted with technical assistance from the CDC using the probability proportional to estimated size (PPES) 30-cluster design, and the reported coverage was validated [16].

### Impact of MDA

A system of sentinel and spot-check sites was set up in the endemic districts in order to evaluate the impact of the MDA campaigns on microfilaremia. Seven sentinel sites were initially selected; due to budget constraints, the number of sites was later trimmed to three sites. Each of these three sites represented a cluster of districts. After the baseline collection in 2000, subsequent sentinel-site activities were conducted before the third and fifth year of MDA in each district. Blood samples were collected from 500 people in each sentinel site between the hours of 22:00 and 02:00. In adherence with WHO guidelines [16], spot-check sites were also selected from the endemic districts as an additional monitoring mechanism prior to the fifth round of MDA using a similar testing methodology to the sentinel sites. Between 2005 and 2009, 13 spot-check sites were conducted from urban sites, cross-border areas, and other locations that were suspected to carry an elevated risk for ongoing transmission.

**Figure 2:**
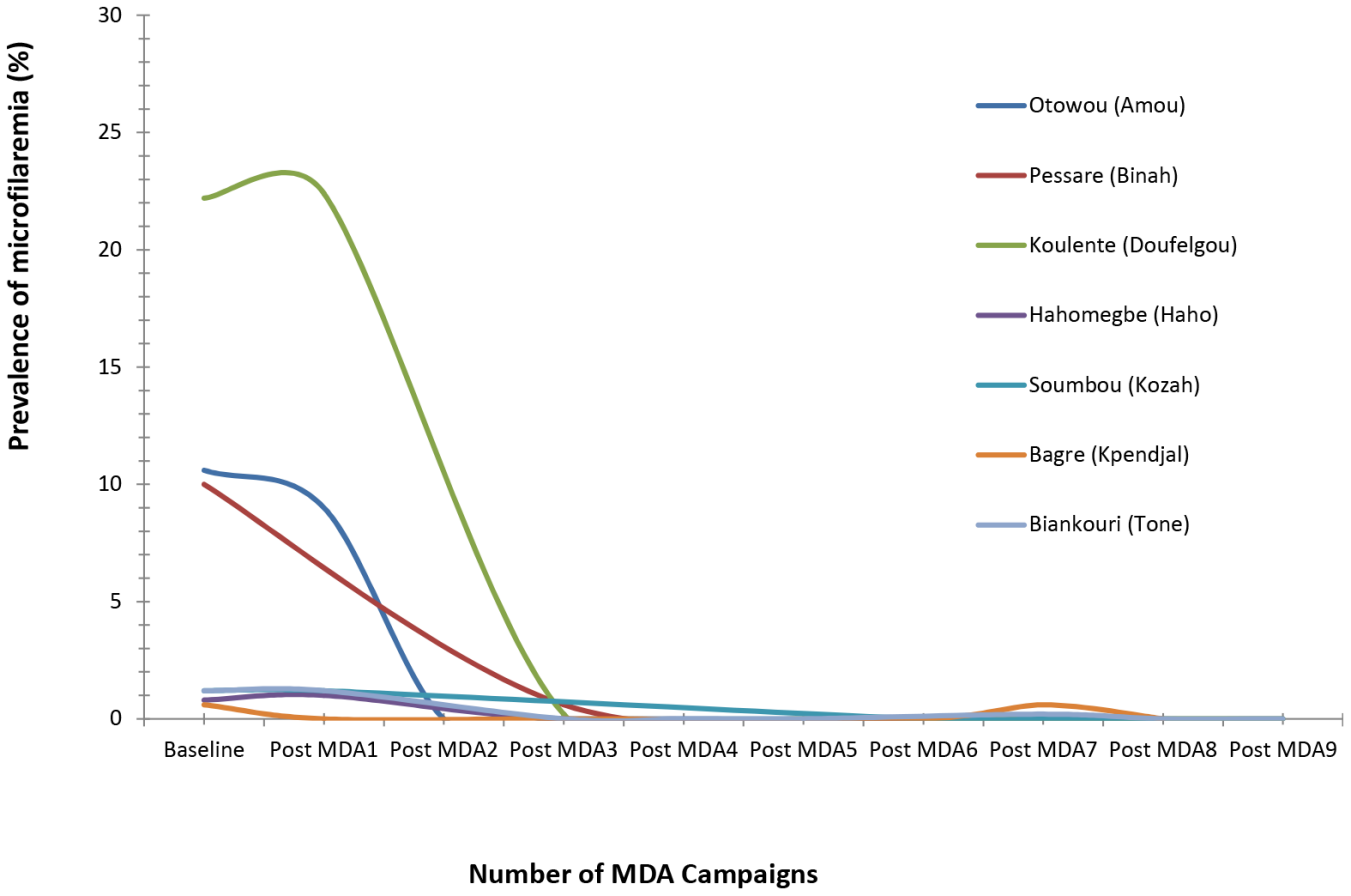
Progression of microfilaremia in sentinel sites in the endemic districts of Togo following subsequent mass drug administrations.

The baseline results from the sentinel sites indicated a microfilaremia prevalence between 1% and 36%. Since 2004, microfilaria samples from the sentinel sites (see Figure 2) and spot checks have all been below 1%. Five of the seven districts have consistently registered prevalence levels of 0% in all sentinel and spot-check sites. MDAs were halted in 2008 following six to seven MDAs. Additional MDA campaigns were conducted in 2009 in two districts: i) in Tone, after seven MDAs, where a prevalence of 2% was detected at the spot-check site of Woriwouri, a village in close proximity to endemic areas in Burkina Faso and Ghana and ii) in Doufelgou, where an additional MDA was organized because, although prevalence level was reported at 0% in the sentinel site after the third MDA, this sentinel village was not assessed after the fifth MDA. No detection of bancroftian filaria was found after the subsequent MDAs. The results from the sentinel and spot-check sites indicate that nine years of MDA campaigns have succeeded in reducing levels of microfilaria in Togo to 0%, thus most likely achieving interruption of transmission.

### Ongoing Surveillance

Togo is the first country to implement a nationwide LF surveillance system. Implemented in 2006, Togo put in place a system to identify and track cases with active infection. Forty laboratories were selected throughout the country for inclusion in surveillance activities. This system utilized nocturnal thick blood smears for malaria diagnosis, as they can concurrently be used to make LF diagnoses [24]. Participation in the surveillance system required laboratory technicians to send all slides with identified *W. bancrofti*, in addition to ten randomly selected slides per month, to the reference laboratory in Lomé.

**Figure 3:**
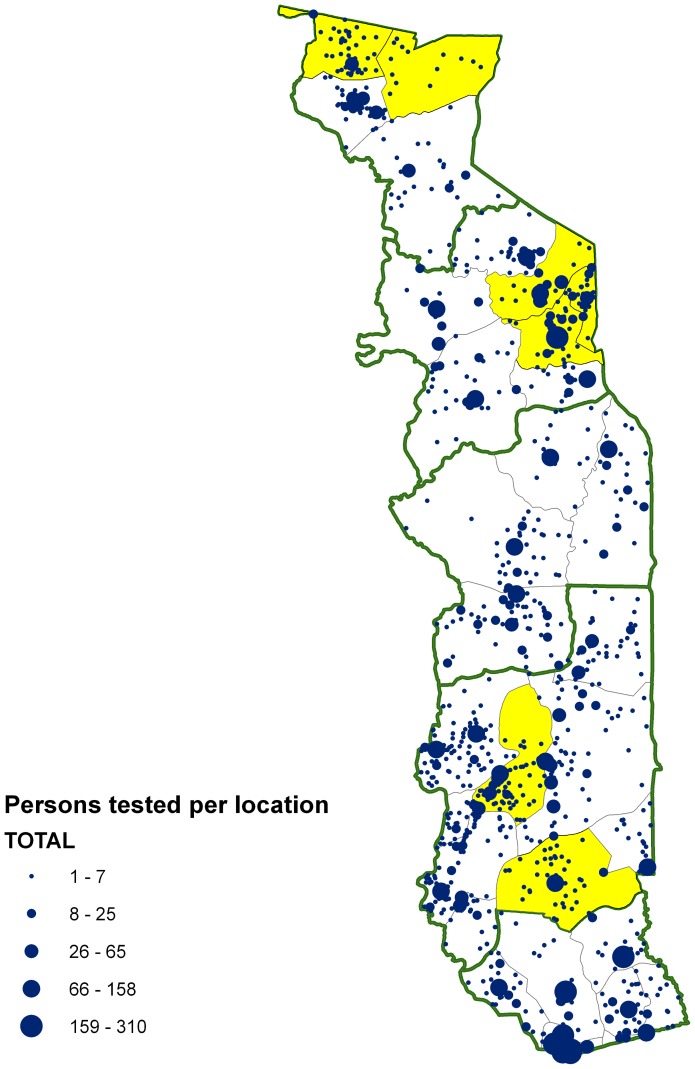
Geographic coverage of the LF surveillance system, 2006–2007.

Within the first four years of the surveillance system, 8,050 slides were accrued by the reference laboratory and two LF-positive slides were identified. Both cases were followed up according to a predetermined algorithm of action, and no further positive cases followed. An evaluation of the system showed that some border areas were not as well covered. It was decided to increase the coverage of the surveillance system by implementation of centralized ELISA-based testing for antigenemia using filter-paper blood spots collected in dispensaries (Figure 3). No active transmission focus has been detected so far by the system.

## Operational Research

Added components that contributed to the success of the NPELF were several operational research projects that further addressed the pillars of the GPELF and expanded program outreach.

### Lymphedema Management Program

As mentioned, operational research implemented with the CDC's assistance resulted in the formation of Togo's national lymphedema program. One of the evaluation tools was a cohort survey (2004–2010) of 188 LF patients to assess impact. The survey addressed indicators such as current and previous treatment practices, quality of life, economic factors, and overall program impact among patients with lymphedema [25]. Between the preprogram assessment in 2007 and the assessment at the end of the funding in 2010, the proportion of patients (n = 107) who did not seek treatment or sought traditional methods decreased from 30.8% to 2.8%. An increase of positive treatment practices was also observed: the practice of cleaning the limb increased from 10.3% to 92.5% and exercising increased from 3.7% to 78.5%, (p<0.0001) (unpublished data).

### Transmission Assessment and Post-treatment Surveillance

As the macrofilaremia in the sentinel sites and spot-check sites were <1%, the NPELF conducted a 30-cluster survey to establish interruption of disease transmission among children two and six years of age. In May 2008, the cluster survey was carried out with support from the Emory University LF Support Center, Atlanta following the WHO 2005 guidelines [16]. ICT card and blood spots for Og4C3 ELISA were performed. The ELISA testing conducted by the CDC indicated that all but one child was negative.

In 2006, the NPELF collaborated with the MDP and the CDC to develop an innovative ongoing national surveillance system. Several sampling modalities, such as using military recruits and blood banks, were considered, but it was decided to base the system on nocturnal thick smears collected to diagnose malaria as described above [24]. This surveillance system was eventually taken over by the MoH as part of the ongoing LF activities. With the financial support of RTI/USAID, the NPELF and the CDC evaluated this system in 2010 and validated also the initial mapping.

In 2012, the first post-MDA transmission assessment surveys (TAS) using the WHO's new guidelines [12] were conducted in the seven districts grouped into four evaluation units (EU). The results suggested that all EUs passed as the number of ICT-positive cases was below the cut-off value of 18. The positive cases included: eight cases in Tone-Kpendjal EU, one case in Binah-Doufelgou EU, four cases in Amouh-Haho EU, and no cases in Kozah EU.

In order to confirm the absence of recrudescence and prepare the country for certification, the post-MDA surveillance activities will continue until 2015. These activities include ongoing surveillance and the implementation of the second and final WHO-recommended TAS scheduled for 2015.

### Other Research

In 2005, the NPELF also contributed to guide the global integrated NTD program by launching a three-year program supported by the CDC. The national NTD program coordinators developed an integrated NTD program during a one-year preparation phase to elaborate integrated tools and guidelines for an integrated NTD program. The integrated areas covered were mapping, baseline data, MDA, health education, and monitoring and evaluation. Thanks to a praziquantel donation from the Schistosomiasis Control Initiative (SCI), the integrated approach was twice piloted in the district of Binah and achieved good results. The coverage rates were high, program managers adhered to the concepts, and chances for sustainability were believed to be strengthened by linking unfunded NTD programs (STH, schistosomiasis) with well-funded programs. Based on this success, the MoH was able to secure funding for a national scale-up.

Furthermore, the NPELF program conducted operational research to validate drug coverage surveys with assistance from the MDP and the CDC. The accuracy of the results of such surveys was tested one, six, and 12 months after an MDA in Togo in which three drugs (albendazole, ivermectin, and praziquantel) were distributed. Survey results indicated that the respondents were able to accurately recall if they took the drugs.

## Advocacy and Fundraising

The main key to success in Togo was the mixture of an opportunistic approach from the NPELF combined with strategic and generous partners. Since the 1990s, Togo has been an underfunded country due to the political climate and subsequent digression from democracy [26], [27]. However, the NPELF was initiated and carried out thanks to financial and technical support from Health & Development International (HDI), a Norwegian NGO which provided support during the first nine years of the program [28]. HDI also made the program visible and attractive to new external partners such as LF program support centers in Atlanta and Liverpool and the CDC. This enabled the NPELF to capitalize on the strength of partnerships and networking and allowed them to implement all the activities required for the elimination of LF. In addition to HDI funding, in 2000, the program was awarded a portion of the DFID grant through the WHO. The collaboration with the onchocerciasis control program facilitated the reduction of the costs of drug distribution and sustained high MDA coverage. The collaboration with the national malaria program resulted in submitting an innovative joint malaria/LF proposal to the Global Fund to Fight AIDS, Tuberculosis and Malaria (GFATM) that secured five years of implementation funding for MDA and impact assessments.

## Discussion

This paper describes not only the first sub-Saharan LF program that probably interrupted transmission, but demonstrates that it is possible and essential for morbidity programs to be codeveloped along with the MDA campaigns in order to fully address the burden of the disease in endemic territories.

The ability to implement an LF national program was not evident due to a variety of embargoes that were placed on Togo in the late 1990s. These embargoes kept the country in a state of severe economic stress. Although in this political climate LF was not perceived as a health priority, the dedication and endorsement of the concept of LF elimination by the MoH has, and continues to be, a critical factor in setting up a successful program. For example, during some years, the program was unable to provide the small MDA incentive of $3 to $6 USD per CHW volunteer. Regardless, the CHWs distributed the medications, motivated by the spirit of the collaborative environment created by the NPELF. Highly motivated CHWs also facilitated high compliance in the communities where LF was either largely unknown or not perceived as a health problem.

A factor contributing to success was the fact that the MoH used existing structures. Indeed, the MDAs for LF elimination were initiated using the preexisting structure of the community-directed ivermectin treatment (CDTI) established by the national onchocerciasis control program [21]. The surveillance and M&E activities in the Togo program required the MoH to engage the decentralized clinical laboratory network to conduct cost-effective impact assessments. It also allowed the MoH to establish robust relationships with research institutions. The NPELF also identified resources within Togo's existing health structure to implement lymphedema and hydrocele management. It was found that the decentralized health system could be used to disseminate lymphedema management training through multiple layers of health staff and community workers.

Additionally, the NPELF demonstrated the ability to recognize and capture each funding opportunity to implement all components of the program. To date, the NPELF remains the only LF program in Africa that has been awarded financial aid from the GFATM. The collaboration with the CDC on several operational research projects enabled the program to implement some crucial aspects of the elimination program such as morbidity management and surveillance. It has also increased the visibility of the program and provided access to even more resources.

It is also important to point out that the impact of this successful program is not limited to only alleviating the filariasis burden in Togo. The program was the base for RTI/USAID funding for an integrated NTD program resulting in finalizing national mapping [29] and implementing national schistosomiasis and STH MDAs. The results of the operational research projects and the lessons learned by the NPELF are widely discussed at international meetings and shared with peers. The NPELF is also assisting other countries in replicating the lymphedema management pilot program through the creation of a national morbidity training manual that describes program components and contains useful information for implementing a national morbidity management program in low-resource settings [30]. This manual is widely shared and is used by other partners such as Handicap International and the CDC Foundation to implement programs in other countries (e.g., Benin and Mali). Based on the Togo experience, surveillance systems will be set up in two additional African countries and the program coordination is providing technical assistance to countries in the region.

The success of the NPELF in Togo shows that lymphatic filariasis can be effectively addressed in countries with limited funds when cooperative efforts combine with motivation and innovation. Ministries of health play a critical role in embracing the scaling up of program activities, and new avenues for partnerships and funding must be vigorously pursued. The main lesson learned is that when developing a national disease elimination strategy, a key component must be putting forth the time and effort to build strong collaborations among appropriate partners. This networking with program collaborators when combined with persistent advocacy and fundraising was the crucial component that permitted the NPELF to address LF in Togo.

In conclusion, as summarized in Table 2, the success of the National Program to Eliminate Lymphatic Filariasis in Togo was facilitated by charismatic, innovative, and trustworthy program managers able to i) timely identify issues and solutions, ii) ensure visibility at the highest levels of the MoH, iii) adopt an opportunistic approach using existing health interventions and a decentralized health system to integrate LF activities, and most importantly, iv) develop a variety of partnerships and pioneering approaches to mobilize resources for a synchronized implementation of the twin-pillar strategies as recommended by the GPELF. The adoption of these approaches could aid other countries that are not on target to reach the 2020 LF elimination goal in sub-Saharan Africa.

**Table 2:** Determinants of success of the National Program to Eliminate Lymphatic Filariasis in Togo.

**1. Strong sustained political commitment**
- Enthusiastic and knowledgeable program coordinators who pushed the agenda at the highest level of the Ministry of Health
- Partners who gave visibility to the program at high level
**2. Small burden of the disease**
- Relatively small size of the country (total area = 56,785 sq km; total population = 6.9 million inhabitants)
- Limited burden (approximately 20% of the total population was at risk)
**3. Establishment of a motivated, knowledgeable management team**
- Strong and trustworthy coordinators who fully understood the program's strategies and challenges and who were fully committed to partnership and innovation
- Coordinators were changed depending on needs of program
- Establishment of a three-prong team (not all full-time)
+ M&E focal person shared with other programs
+ lymphedema management focal person
+ hydrocele management focal person (surgeon, part-time adviser)
**4. Flexible administration**
- Easy access of the program coordinator to the highest level of the Ministry of Health
- Administration was willing to adapt to program's needs: flexible system to obtain travel orders, money from the account
**5. Integration with existing health interventions**
- Easy access of the program coordinator to the highest level of the Ministry of Health
**6. Innovative resources mobilization in an environment totally lacking local resources**
- Jumped on all opportunities to get free health education emissions by the national television and local radios
- Joint malaria/LF proposal granted by the GFATM
- Accepted small research funds ($10,000 USD or up) to start innovative program
**7. Very strong partnerships built up starting from no existing partnerships**
- Internal partnerships (with community drug distributors, district health team, academic institutions, etc.)
- External partnerships (with GSK, Merck, MDP, HDI, LF support centers of Atlanta and Liverpool, CDC, IMA, GFATM)

## Way Forward

In agreement with the new guidelines of the WHO [12], in 2010, the NPELF of Togo entered into a five-years post-MDA surveillance phase that will take the country to the WHO's independent verification of absence of the transmission in 2015. During this surveillance phase, the lab-based surveillance will continue throughout the country with an emphasis on areas bordering endemic districts of the three neighboring countries where the MDAs are still ongoing. In addition, the final TAS will be implemented in 2015 in the seven previously identified endemic districts. The current challenge is to maintain momentum for adequate human and financial resources allocation in order to support the implementation of the critical endgame activities. The program coordination is closely working with the national malaria control program in order to reach/maintain universal insecticide-treated net coverage. Most importantly, it is the desire to increase the net compliance rate that was demonstrated to be a problem in the Togolese communities [31]. The morbidity control activities are expected to be fully integrated within the health system and are deemed to continue providing care to patients even beyond the verification target.
